# Fourth ventricular solitary fibrous tumor: a case report and review of the literature

**DOI:** 10.1186/1752-1947-6-205

**Published:** 2012-07-17

**Authors:** Congli Wang, Varsha Manucha, Scott Faro, Michael Weaver, Abir L Mukherjee

**Affiliations:** 1 Department of Pathology and Laboratory Medicine, Temple University Hospital, 3401 North Broad Street, Philadelphia, PA, 19140, USA; 2 Department of Radiology, Temple University Hospital, Philadelphia, PA, USA; 3 Department of Neurosurgery, Temple University Hospital, Philadelphia, PA, USA

**Keywords:** Fourth ventricle, Intraventricular, Solitary fibrous tumor

## Abstract

**Introduction:**

Solitary fibrous tumors of the central nervous system usually present as dura-based masses and clinically resemble meningiomas. There are very few reported cases of intra-ventricular solitary fibrous tumors, particularly in the fourth ventricle.

**Case presentation:**

Our patient was a 52-year-old African-American man, who presented to our facility with a two-month history of progressive weakness and numbness in all extremities. A computed tomography scan and brain magnetic resonance imaging scan revealed a homogeneous, avidly enhancing 4.5 × 3.7 × 2.7cm fourth ventricular mass, with compression of adjacent medulla and cerebellum and extension into the foramen of Luschka. Our patient underwent a suboccipital craniotomy and resection of the tumor. A histological examination showed a spindle cell neoplasm with prominent collagenized stroma. The neoplastic cells were strongly and diffusely positive for CD34, vimentin and Bcl-2, and negative for S-100, CD99 and epithelial membrane antigen. The molecular immunology Borstel-1 (MIB-1) proliferation index was low (1%). CD31 immunostain highlighted the endothelial cells but the spindle cells were negative. Reticulin stain demonstrated a moderate reticulin network but individual cells were not invested by reticulin fibers. The histological features and immunoprofile was consistent with a solitary fibrous tumor.

**Conclusions:**

In the central nervous system, solitary fibrous tumors are usually indolent tumors, with only rare examples showing hypercellularity and increased mitotic activity; features that were absent in our patient’s case. We present an uncommon central nervous system neoplasm in a rare location. Although uncommon, solitary fibrous tumors should be included in the differential diagnosis of intra-ventricular tumors in adults.

## Introduction

Solitary fibrous tumors (SFTs) were originally described in pleura and subsequently in soft tissue and many other organs including the central nervous system. SFTs of the central nervous system are rare entities, usually presenting as dura-based masses predominantly in the posterior fossa and spinal region [1]. SFTs are composed of spindle cells in a collagenized stroma with characteristic immunohistochemical features: diffuse and strong reactivity to CD34, positive for vimentin, and often immunoreactive to CD99 and Bcl-2 [1]. Intra-ventricular SFTs are rare, and only six cases have been reported in the fourth ventricle [2-7].

## Case presentation

**Figure 1:**
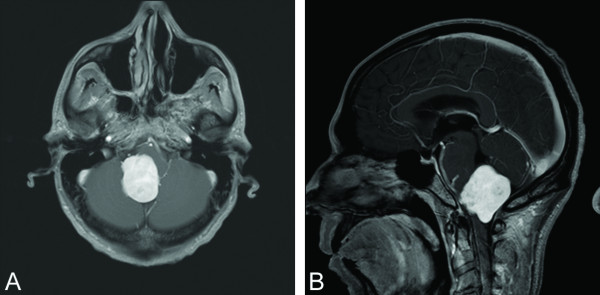
Contrast-enhanced T1-weighted axial (A) and sagittal (B) magnetic resonance imaging scans showing a homogenous mass located in the fourth ventricle.

Our patient was a 52-year-old African-American man who presented to our facility with a two-month history of progressive weakness and numbness in all extremities. A computed tomography (CT) scan and a brain magnetic resonance imaging (MRI) scan revealed a 4.5 × 3.7 × 2.7cm, homogeneous, avidly enhancing mass located in the fourth ventricle, compressing adjacent medulla and cerebellum and extending into the foramen of Luschka (Figure 1). Angiography demonstrated multiple dysplastic-appearing branches from the right posterior inferior cerebellar artery supplying the neoplasm.

Our patient underwent a suboccipital craniotomy and resection of the tumor. During the operation, a cavitron ultrasonic surgical aspirator as well as bipolar cautery and suction were used to debulk the tumor, followed by a combination of RHOTON™ dissectors as well as microscissors to separate the tumor. The entire tumor was successfully removed.

**Figure 2:**
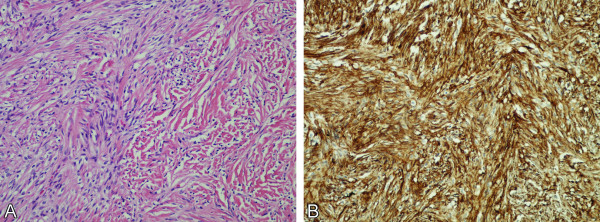
**Photomicrographs (original magnification, 200×).** (**A**) Bland spindle cell tumor with collagenized stroma. (**B**) Neoplastic cells are strongly immunoreactive to CD34.

**Figure 3:**
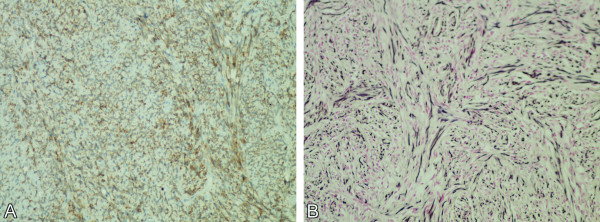
**Photomicrographs (original magnification, 200×).** (**A**) The tumor cells are immunoreactive to Bcl-2. (**B**) Neoplastic cells have moderately rich reticulin network.

Histological examination showed a spindle cell neoplasm with prominent collagenized stroma (Figure 2A). Mitotic figures were not readily found and the molecular immunology Borstel-1 (MIB-1) proliferation index was low (1%). Meningothelial whorls and psammoma bodies were absent. The neoplastic cells were strongly and diffusely positive for CD34 (Figure 2B), vimentin and Bcl-2 (Figure 3A); no immunoreactivity with S-100, CD99 and epithelial membrane antigen (EMA) was detected. A CD31 immunostain highlighted the endothelial cells but not the spindle tumor cells. A moderate reticulin network was demonstrated by reticulin staining, but individual cells were not invested by reticulin fibers (Figure 3B). The histological features and immunoprofile were consistent with SFT.

Our patient experienced no neurological deficit after surgery, and there were no signs of recurrence at one-year follow-up.

## Discussion

Intraventricular tumors can have a wide spectrum of differential diagnosis based on radiological and histopathological features. A bland spindle cell tumor with collagenized stroma and avid contrast enhancement initially suggested a diagnosis of fibroblastic meningioma. However the immunoprofile of the neoplastic cells was typical of a solitary fibrous tumor (negative for EMA, strongly positive for CD34, vimentin and Bcl-2). In contrast, meningiomas are EMA positive and CD34 negative. Hemangiopericytomas can have histological overlap with solitary fibrous tumor, but CD34 and Bcl-2 staining is weak and patchy [8].

To the best of our knowledge, just 17 Intraventricular solitary fibrous tumors have been reported in the literature to date. Six of these tumors were located in the fourth ventricle [2-7], eight cases were in lateral ventricle location [1,9-14], one tumor located in the third ventricle [15], one case occurred in the foramina of Monro [16] and one case was a recurrent SFT arising from the falx cerebri, extending to the bilateral lateral ventricles and the third ventricle [17]. The clinical and radiological features of these tumors are given in Table 1; the immunomorphological features are given in Table 2. The age of patients ranged from 11 to 75 years old, with the vast majority being over 40 years; there was no sex predominance. Based on the available data, all the tumors were less or equal to 5.0 cm in their greatest dimension. On MRI scans, the tumors are typically iso- to hypointense on T1-weighted images, and show intense and homogeneous enhancement after intravenous administration of gadolinium. Most of those SFTs had bland histological features except two tumors showed mild to marked pleomorphism [5,11]. Intraventricular SFTs have an indolent clinical course and excellent prognosis after surgery; however, owing to the small number of cases and limited follow-up, more data is needed to better assess the biological behavior of these tumors and the long-term outcome after treatment.

**Table 1:** Summary of clinical and radiological features of intraventricular solitary fibrous tumors reported in the literature

**Reference**	**Age/sex**	**Location**	**Size (greatest dimension)**	**Imaging features**	**Follow-up**
Clarençon *et al*. [2]	32/F	Fourth ventricle	2.5cm	MRI: hypointense T1; heterogeneous- hypointense T2; post-contrast enhancement	NA
Cummings *et al*. [3]	52/M	Fourth ventricle	NA	MRI: homogeneous post-contrast enhancement	Autopsy
Gessi *et al*. [4]	63/F	Fourth ventricle	2cm	MRI: isointense T1; partial post-contrast enhancement	NA
Kim *et al*. [5]	49/F	Fourth ventricle	NA	MRI: dense post-contrast enhancement	No recurrence after one year
Montano *et al*. [6]	61/M	Fourth ventricle	NA	MRI: hypointense T1; iso-hypointense T2; marked post-contrast enhancement	No recurrence after two years
Sawauchi *et al*. [7]	57/M	Fourth ventricle	NA	MRI: low intensity T1; homogeneous post-contrast enhancement	NA
Tihan *et al*. [1]*	NA	Lateral ventricle	NA	MRI: low density T1; post-contrast enhancement	NA
Clarençon *et al*. [9]	44/F	Right lateral ventricle	4.0cm	MRI: hypointense T1; hyperintense T2; thin peripheral enhancement of multiple confluent cysts post-contrast	NA
Liao *et al*. [10]	NA	Right lateral ventricle	NA	NA	NA
Mekni *et al*. [11]	40/M	Right lateral ventricle	3.5cm	NA	No recurrence after three years
Surendrababu *et al*. [12]	55/F	Left lateral ventricle	5.0cm	NA	No recurrence after one year
Vassal *et al*. [13]	60/F	Left lateral ventricle	5.0cm	MRI: lobulated mass; homogeneous post-contrast enhancement	No recurrence after two years
Wright *et al*. [14]	11/F	Right lateral ventricle	NA	NA	NA
Koçak *et al*. [15]	63/M	Third ventricle	2.5cm	MRI: isointense T1 and T2; significant post-contrast enhancement	No recurrence after three and a half years
Kinfe *et al*. [16]	75/F	Foramen of Monro	2.5cm	CT: Hypointense tumor at the foramina of Monro; homogeneous post-contrast enhancement	No recurrence after one year
Teranishi *et al*. [17]	61/M	Falx cerebri, extended to bilateral lateral ventricles and the third ventricle	NA	MRI: isointense T1; iso-hyperintense T2; heterogeneous post-contrast enhancement	NA
Present case	52/M	Fourth ventricle	4.5cm	Iso-hypointense T1; heterogeneous T2; homogeneous post-contrast enhancement	No recurrence after one year

*Two cases.

NA = not available.

MRI=magnetic resonance imaging.

**Table 2:** Summary of immunomorphological features of intraventricular solitary fibrous tumors reported in the literature

**Reference**	**Histologic features**			**Immunohistochemical features**						
	**Pleomorphism**	**Mitosis**	**Necrosis**	**CD 34**	**Bcl-2**	**Vimentin**	**S-100**	**EMA**	**MIB-1**	**Reticulin**
Clarençon *et al*. [2]	NA	NA	NA	NA	NA	NA	NA	NA	NA	NA
Cummings *et al*. [3]	NA	NA	NA	Strong, diffuse **+**	Strong, diffuse **+**	Strong, diffuse **+**	**-**	**-**	NA	NA
Gessi *et al*. [4]	NA	Absent	Absent	Strong **+**	Strong **+**	Strong **+**	**-**	**-**	<2%	NA
Kim *et al*. [5]	Mild	Rare	NA	Diffuse **+**	NA	NA	**-**	**-**	<3%	Outlining of individual cells **-**
Montano *et al*. [6]	NA	NA	NA	**+**	**+**	**+**	**-**	**-**	<1%	NA
Sawauchi *et al*. [7]	NA	NA	NA	**+**	NA	**+**	NA	NA	NA	NA
Tihan *et al*. [1]*	NA	NA	NA	Strong **+**	NA	Strong **+**	**-**	**-**	NA	Overall highlighted only the coarse collagen background
Clarençon *et al*. [9]	NA	NA	NA	Strong **+**	NA	NA	NA	NA	NA	NA
Liao *et al*. [10]	NA	NA	NA	NA	NA	NA	NA	NA	NA	NA
Mekni *et al*. [11]	Marked	5/10 HPF	Focal	Strong **+**	**+**	Strong **+**	NA	NA	<2%	NA
Surendrababu *et al*.[12]	Absent	NA	NA	**+**	**+**	**+**	**-**	**-**	<2%	NA
Vassal *et al*. [13]	Absent	Absent	Absent	Diffuse **+**	NA	Diffuse **+**	**-**	**-**	<2%	NA
Wright *et al*. [14]	NA	NA	NA	NA	NA	NA	NA	NA	NA	NA
Koçak *et al*. [15]	NA	NA	NA	Focal **+**	Diffuse **+**	NA	**-**	**-**	1%	Interlacing reticular fibers **+**
Kinfe *et al*. [16]	NA	NA	NA	Strong **+**	Strong **+**	Strong **+**	**-**	**-**	<1%	Rich network of reticular fibers
Teranishi *et al*. [17]	NA	NA	NA	Strong **+**	NA	**+**	**-**	**-**	5%	NA
Present case	Absent	Absent	Absent	Strong, diffuse **+**	Strong, diffuse **+**	Strong, diffuse **+**	**-**	**-**	1%	Reticulin network moderate **+**; individual cells **-**

*Two cases.

HPF = high power field; NA = not available; + = positive; **- =** negative.

## Conclusions

We report a rare case of SFT in an unusual location. Though rare, SFTs should be considered in the histological differential diagnosis of intra-ventricular tumors particularly, spindle cell tumors with collagenized stroma, along with meningioma and schwannoma.

## Consent

Written informed consent was obtained from the patient for publication of this manuscript and any accompanying images. A copy of the written consent is available for review by the Editor-in-Chief of this journal.

## Competing interests

The authors declare that they have no competing interests.

## Authors’ contributions

CW performed the gross examination of the specimen, conceived the case report, searched the literature and drafted the manuscript. VM performed the histopathological evaluation of the slides and made substantial revisions to the manuscript. SF conducted the radiology examinations and made revisions to the manuscript. MW operated on our patient and made revisions to the manuscript. AM performed the histopathological evaluation of the slides and critical analyzed the manuscript. All authors read and approved the final manuscript.
